# Neighbourhood effect and inequality in access to essential health services among mother–child paired samples: a decomposition analysis of data from 58 low- and middle-income countries

**DOI:** 10.1186/s12939-024-02194-4

**Published:** 2024-06-28

**Authors:** Seun Anjorin, Elvis Anyaehiechukwu Okolie, Chinwe Onuegbu, Mukhtar Ijaiya, Abimbola Ayorinde, Oyinlola Oyebode, Olalekan Uthman

**Affiliations:** 1 Division of Health Sciences, University of Warwick, Coventry, UK; 2 Big Data Institute, Nuffield Department of Population Health, University of Oxford, Oxford, UK; 3 Department of Public Health, David Umahi Federal University of Health Sciences, Uburu, P.M.B 211 Nigeria; 4 School of Health and Life Sciences, Teesside University, Middlesbrough, UK; 5 Jhpiego, Plot 971, Rueben Okoya Crescent, Off Okonjo Iweala Street, Wuye District, Abuja, FCT Nigeria; 6 NIHR Applied Research Collaboration West Midlands, Warwick Medical School, University of Warwick, Coventry, UK; 7 Centre for Public Health & Policy, Wolfson Institute of Population Health, Barts and The London School of Medicine and Dentistry, Queen Mary University of London, London, UK; 8 Oxford Brookes University, Oxford, UK

## Abstract

**Introduction:**

Neighbourhood effect on health outcomes is well established, but little is known about its effect on access to essential health services (EHS). Therefore, this study aimed to assess the contributing factors to access to EHS in slum versus non-slum settings.

**Methodology:**

The most recent data from 58 Demographic and Health Surveys (DHS) conducted between 2011 and 2018 were used, including a total of 157,000 pairs of currently married women aged 15–49 and their children aged 12–23 months. We used meta-analysis techniques to examine the inequality gaps in suboptimal access to EHS between mother-children pairs living in slums and non-slums. Blinder-Oaxaca decomposition technique was used to identify the factors contributing to the inequality gaps in each low- and middle-income country (LMIC) included.

**Result:**

The percentage of mother–child pairs living in slums ranged from 0.5% in Egypt to 63.7% in Congo. Meta-analysis of proportions for the pooled sample revealed that 31.2% [27.1, 35.5] of slum residents and 20.0% [15.3, 25.2] among non-slum residents had suboptimal access to EHS. We observed significant pro-slum inequalities in suboptimal access to EHS in 28 of the 52 LMICs with sufficient data. Of the 34 African countries included, 16 showed statistically significant pro-slum inequality in suboptimal access to EHS, with the highest in Egypt and Mali (2.64 [0.84–4.44] and 1.76 [1.65, 1.87] respectively). Findings from the decomposition analysis showed that, on average, household wealth, neighbourhood education level, access to media, and neighbourhood-level illiteracy contributed mostly to slum & non-slum inequality gaps in suboptimal access to EHS.

**Conclusion:**

The study showed evidence of inequality in access to EHS due to neighbourhood effects in 26 LMICs. This evidence suggests that increased focus on the urban poor might be a important for increasing access to EHS and achieving the universal health coverage (UHC) goals.

**Supplementary Information:**

The online version contains supplementary material available at 10.1186/s12939-024-02194-4.

## Background

The term “Neighbourhood effect” describes the likelihood that the environment where people live and work influences people’s life experiences and health outcomes [1–3]. This concept takes its root in the works of one of the early scholars of public health John Snow, on the cholera outbreak in London in the 19th century [4]. Unlike the popular opinion in an era where diseases like cholera were considered airborne, Snow’s mapping showed that cholera was linked to people drinking from a contaminated water pump in an area [4].

Snow’s findings heralded a change of concepts in the epidemiology of diseases and influenced an increased focus on the impact of neighbourhoods on health outcomes and the need for improved sanitation [5, 6]. Also, it is believed that the emergence of multilevel methodological analysis can advance the publication of empirical studies in this field, thus highlighting the importance of the neighbourhood on health. It has since become a main aspect of public health research as previous studies have focused on neighbourhood effects on health outcomes ranging from morbidity to mortality in different populations and contexts [7–10].

While neighbourhood effects occur in different settings, their association with health outcomes in slums is particularly important. Slums are densely populated low-resource settings in urban areas [11]. Environmental factors in slums, including poor water, sanitation and hygiene, and lack of infrastructure are likely to affect the collective health of slum residents. Likewise, interventions done in slums might benefit many at once [12].

This study builds on our previous study which explored the multilevel determinants of access to Essential Health Services (EHS) in 58 LMICs [13]. One of the key findings from the study was that the neighbourhood effect, as measured by intra-class correlation (ICC) and variance partition correlation (VPC), is strongly associated with mother–child pair access to EHS. In this study, we use slums in LMICs to conceptualise neighbourhood effects and perform further analyses to examine the inequality in access to EHS due to neighbourhood effects.

## Methods

### Data source, study design & selection

We used nationally representative data from the demographic and health surveys (DHS) based on a cross-sectional research design [14]. Historically, the DHS was focused on reproductive and fertility data but has since expanded to cover indicators relating to nutrition, household characteristics, and maternal and child outcomes. The DHS is primarily funded by the US Agency for International Development (USAID) in collaboration with the national agencies in charge of population census for each LMIC. Therefore, they have large sample sizes, usually between 5,000 and 30,000 households across 90 LMICs, and are conducted every five years to allow comparisons over time. The survey utilises a stratified two-stage cluster design. Enumeration areas (EA) are drawn from a census file; the second stage involves drawing a sample of households from an updated list of households in selected EA. The sample is generally representative at the national, regional, and residential levels. Details of the data collection procedure have been published elsewhere [15].

We selected dyads of women aged 15-49yrs, and their children aged 12–23 months, born within 5 years before the survey as our unit of analysis. The selection of countries was carried out in two stages. In the first stage, we reviewed data from all recent surveys from LMICs conducted between 2010 and 2018 to determine if the survey collected information on UHC essential health service indicators. In the second stage, we selected the most recent DHS survey in countries with more than one survey within the period. Based on data availability from each country, we included 58 LMICs in this study. We believe this will allow evidence generation from diverse geographical regions with different health sector designs, varying levels of resource investment, diverse socio-cultural features, and different patterns of exposure and outcome.

### Outcome – access to EHS

Suboptimal access to EHS is the primary outcome variable computed from nine universal health coverage (UHC) essential health service indicators as recommended in the recent monitoring framework from the WHO and the World Bank [16]. We used the following indicators based on their availability across the selected countries: access to family planning for the child’s mother, four antenatal visits (ANC), BCG immunisation, three doses of DPT3 immunisation, measles immunisation, and the use of insecticide-treated nets (ITN), skilled birth attendance, oral hydration treatment (ORT) for childhood diarrhoea and acute respiratory infection treatment for childhood pneumonia (See Table 1 for their definitions). We defined the outcome variable as a binary variable; suboptimal access if a mother (aged 15–49 years) and child (aged 12–23 months) pair has received three or fewer of the nine indicators and optimal access if the mother–child pairs had access to more than three of the nine indicators. These cut-off points were determined based on explorations of the dataset by computing the interquartile range (IQR) for access to EHS; the lowest was 3. A similar cut-off point was also used in the most recent and relevant study on the global monitoring report on UHC [16].

**Table 1:** Description of DHS data by countries and prevalence of suboptimal access to EHS among mother–child pairs living in urban slums and non-slums

Country	Year	No of Mother–child pair	Mother–child pairs living in slums	Suboptimal Access to EHS (%)	Slum residents with suboptimal access to EHS (%)	Non-slum residents with suboptimal access to EHS (%)
Afghanistan	2015	1,462	25.5	38.9	41.8	37.9
Albania	2018	216	5.6	59.4	91.7	58
Angola	2016	1,198	33.1	38.9	48.1	34.3
Bangladesh	2014	511	20.2	31	39.8	28.3
Benin	2018	952	46.3	24.4	32.9	16.3
Burkina Faso	2010	625	11.4	24.4	26.8	24.1
Burundi	2017	365	14.0	17.8	19.6	17.3
Cambodia	2014	391	11.0	9.5	20.9	6.8
Cameroon	2011	796	18.8	20.1	38.7	16
Chad	2015	575	75.7	49.9	51.7	40.9
Colombia	2016	1,130	4.0	100	100	100
Comoros	2012	221	37.1	32.1	30.5	33.6
Congo DR	2014	383	60.8	24.7	32.9	12.8
Congo	2012	945	63.7	7.6	9.4	6.9
Cote d'Ivoire	2012	440	10.5	25.1	50	22.7
Dominican Republic	2013	391	3.1	20.2	16.7	20.2
Egypt	2014	1,353	0.5	10.8	57.1	10.5
Ethiopia	2016	397	34.3	35.9	52.2	15.2
Gabon	2012	528	13.3	43.6	51.4	42.5
Gambia	2013	493	6.9	11.3	14.7	11.2
Ghana	2014	416	10.1	8.7	14.3	7.9
Guatemala	2015	674	15.0	17.9	34.7	15
Guinea	2018	388	4.9	33.9	42.1	33.5
Haiti	2017	318	24.8	33.8	51.9	28.6
Honduras	2012	593	17.4	5.1	1	5.8
India	2016	12,018	20.2	19.3	25.8	18
Indonesia	2017	1,744	2.6	24	39.1	23.5
Kenya	2014	1,111	28.3	10.4	14.3	8.9
Lesotho	2014	133	6.0	12.5	12.5	12.3
Liberia	2013	345	73.6	17.6	24	10.4
Malawi	2016	445	2.7	9.4	8.3	9.5
Mali	2018	494	7.5	10.9	16.2	10.1
Mozambique	2011	609	40.9	21.2	24.5	18.4
Myanmar	2016	203	49.3	11.8	20	4.4
Namibia	2013	220	31.4	17.3	18.8	16.7
Nepal	2016	577	35.5	20.8	28.3	16.8
Niger	2012	516	9.9	16	23.5	14.6
Nigeria	2018	2,109	30.7	26.3	38.1	22
Pakistan	2018	1,101	5.4	23.7	45.8	21.8
Peru	2012	943	22.2	17.6	23.4	16
Philippines	2017	630	10.2	22.7	46.9	20.6
Rwanda	2015	299	11.0	5.9	15.2	4.5
Senegal	2017	746	5.2	7.3	17.9	6.7
Sierra Leone	2013	532	27.4	23.1	26.7	21.1
South Africa	2016	152	9.2	26.4	42.9	24.9
Tajikistan	2017	393	10.9	17.6	25.6	16.4
Tanzania	2016	416	14.2	10.6	11.9	10.3
Timor-Leste	2016	417	1.9	18.9	37.5	18.6
Togo	2014	405	9.6	28.9	28.2	29.1
Uganda	2016	488	30.7	10.6	15.3	9
Yemen	2013	705	18.7	37.8	53	34.8
Zambia	2014	735	23.9	25.3	26.7	25
Zimbabwe	2015	340	4.1	19.6	42.9	18.7

### Exposure – slum and its classification

The DHS’s main geographical identifier is the region where study participants live, corresponding to each country’s administrative province or state. In addition to this, the dataset from the DHS also captures the residential type of respondents – rural or urban areas. Slum is a relative concept linked to people’s standard of living, however, the perception of slum is generally underpinned by two things – “urban poor” and “informal settlement” [17]. According to the United Nation’s Habitat [11], slum residence is defined as dwelling in a household with one of the following characteristics;

- Poor or inadequate access to improved water
- Poor or insufficient access to improved sanitation
- Poor quality of housing or home structure
- Overcrowded household
- Inability to secure tenure status or predisposed to evictions.

Although these five elements are broad, defining slum households as one with only one of the characteristics will lead to high numbers of slum dwellers; for example, lack of basic sanitation is highly prevalent in LMICs. Also, this definition identifies households only, thus lacking the spatial clustering dimensions associated with living in a slum neighbourhood and associated health outcomes. An alternative method to define slums was adopted, which combines the first four characteristics from the UN-Habitat definition above. Due to the unavailability of data, the last characteristic (“inability to secure tenure status”) could not be operationalised.

Neighbourhood-based concept of slum dwellers was operationalised using DHS data by following a similar approach by Gunther and Harttgen in their seminal paper [17]. The cluster sampling approach by the DHS makes this achievable as each census EA has clearly defined boundaries with numbers of households. Therefore, households sampled within the same census EA are usually within the same neighbourhood. Considering that the interest of the study is in the health condition of the poorest neighbourhood in each country, extreme coding rules were adopted in the analysis. Therefore, mother–child pairs that meet the two conditions below were classified as slum dwellers:

- Living in a household characterised by a minimum of two of the four UN-Habitat characteristics.
- Living in household clusters or neighbourhoods in which the majority (minimum of 50%) of households are defined by two or more of four UN-Habitat characteristics.

Only urban respondents were included in the analysis to reduce the complexity and possible miss-classifications associated with the use of respondents from rural areas and towns. Therefore, mother–child pairs that could not meet the two conditions above were coded zero (i.e. non-slum dwellers).

### Covariates

As informed by previous research on UHC, several demographics, socioeconomic and environmental covariates at the individual level were included in the analysis. The following individual-level variables were included, mother’s age, maternal age of marriage and religion, child’s age, and child’s gender. Other individual-level variables included in the models are the mother’s employment status, access to health insurance, access to media (TV, radio and newspapers), and household headed by a female.

### Statistical analyses

A total of 47,112 mother–child pairs data nested in 1,362 neighbourhoods from 58 LMICs who took part in DHS between 2011 and 2018 were analysed. Descriptive and analytical analyses, univariable and bivariable analyses were performed to show the distribution of DHS respondents used in this study. The weighted prevalence of mother–child pairs living in slums and those with suboptimal access to EHS were computed; meta-analysis of proportion was used to examine the pooled prevalence across the 58 LMICs. Estimates were expressed as percentages, mean and standard deviation. Individual participant meta-analysis was performed to explore the risk difference (RD) of suboptimal access to EHS between mother–child pairs dwelling in urban slums and those otherwise. Urban pro-slum inequality in suboptimal access to EHS occurs among mother–child pairs when the RD is greater than zero. Non-slum inequalities occur RD is less than (i.e. negative); it showed that suboptimal access to EHS is more prevalent among those not living in slums. A random effect model was used to examine the overall RD among all mother–child pairs irrespective of their countries and a map to display the pattern of RD in suboptimal access to EHS.

A variant of Blinder-Oaxaca’s decomposition analysis was performed. Theoretically, this analytical technique uses counterfactual regression equations to construct an assumption that study participants from two groups of exposure (urban slum and urban non-slum dwellers as in this study) have similar measurable characteristics. The technique will divide the differential of the dependent variable (e.g. suboptimal access to EHS) between the two groups into parts. One part will explain how much of the difference is accounted for by the difference in the distribution of included independent variables. This is called the compositional or explained component of the inequality gap). The second part is the unexplained portion (structural component) that captures gaps due to the differential in regression coefficient and variables not included or measured. Due to having a binary outcome variable in this study, the non-linear variant of the Oaxaca-Blinder decomposition technique called the Farlie model was applied for non-linear binary outcomes to build the models. This approach assumes that the conditional expectation of the likelihood that a mother–child pair has suboptimal access to EHS is a non-linear function of a vector of characteristics [18]. All the analyses performed were weighted using the sample weights; this was to make the data more representative of each country, hence, the generalisation of findings could be assumed.

## Results

### Descriptive

Table 1 shows the weighted descriptive statistic of data used by each country; it involves the year of the survey, the total number of mother–child pairs, the prevalence of suboptimal access to EHS and of mother–child pairs residing in urban slum and non-slum but with suboptimal access to EHS. The percentage of slum residents ranged from 0.5% in Egypt to 63.7% in Congo. The weighted average of suboptimal access to EHS was 23.4%, ranging from 5.1% in Honduras to 100% in Colombia (excluded from other analyses due to insufficient data to compute meta-analysis).

Table 2 provides additional descriptive statistics with the pooled sample across the included independent covariates. About 56% of mothers married after their 18th birthday and 53.7% were aged between 25–34 in the survey. Also, 38.9% were not working, 11.4% had no access to media (27.8 residing in urban slums as against 7.2 in non-slums), and only 15.6% had access to health insurance. Also, the prevalence of poor households was 22.4% among urban slum dwellers compared to 5.7% among non-slum dwellers; 7.8 residing in urban slums had access to health insurance while 18.0% of urban non-slum dwellers had. If a woman heads a household, the mother’s employment status in urban slums and non-slum were relatively the same.

**Table 2:** Descriptive statistics with the pooled sample of characteristics of DHS data in LMICs

	Overall	Non-slum residence	Slum residence	*P* test
	47112	36380	10732	
**Age of marriage = > = 18 (%)**	26667 (56.6)	22285 (61.3)	4382 (40.8)	< 0.001
**Maternal age (%)**				< 0.001
13–24	14520 (30.8)	10737 (29.5)	3783 (35.2)	
25–34	25311 (53.7)	20101 (.3)	5210 (48.5)	
35–49	7281 (15.5)	5542 (15.2)	1739 (16.2)	
Maternal education (%)				< 0.001
No education	8926 (19.2)	5285 (14.8)	3641 (34.4)	
Primary	9298 (20.0)	6347 (17.7)	2951 (27.9)	
Secondary education & higher	28179 (60.7)	24184 (67.5)	3995 (37.7)	
**Wealth (%)**				< 0.001
Low	4465 (9.5)	2059 (5.7)	2406 (22.4)	
Middle	10442 (22.2)	6613 (18.2)	3829 (35.7)	
High	32205 (68.4)	27708 (76.2)	4497 (41.9)	
**Media access (%)**				< 0.001
No access	5375 (11.4)	2603 (7.2)	2772 (25.8)	
At least one	15217 (32.3)	11434 (31.4)	3783 (35.2)	
At least two	16177 (34.3)	13326 (36.6)	2851 (26.6)	
All the three	10343 (22.0)	9017 (24.8)	1326 (12.4)	
**Maternal Female-head = 1 (%)**	6603 (14.0)	5010 (13.8)	1593 (14.8)	0.005
**Maternal Not-working = 1 (%)**	18322 (38.9)	14412 (39.6)	3910 (36.4)	< 0.001
**Maternal had health insurance = 1 (%)**	7372 (15.6)	6531 (18.0)	841 (7.8)	< 0.001
**Money problem in accessing health (%)**	14812 (34.1)	10084 (30.0)	4728 (48.2)	< 0.001

As shown in Table 3, meta-analysis of proportions for the pooled sample revealed that 31.2% [27.1, 35.5] of slum dwellers had suboptimal access to EHS compared to 20.0% [15.3, 25.2] among non-slum dwellers.

**Table 3:** D**e**scriptive result showing from the meta-analysis of proportion, mean and interquartile range (IQR) of pooled sample for key variables

**Key variables**	Meta-proportion	Mean (SD)	IQR
Suboptimal access to essential health services	23.4 [25.1, 34.3]	30.4[18.3]	21.9
Suboptimal access to EHS mong mother–child pairs dwelling in slums	31.2 [27.1, 35.5]	31.3[23.1]	23.0
Suboptimal access to EHS mong mother–child pairs dwelling in non-slums	20.0 [15.3,25.2]	20.1 [16.3]	9.9

### Magnitude of sub-optimal access to EHS in urban slums and non-slum

**Fig. 1:**
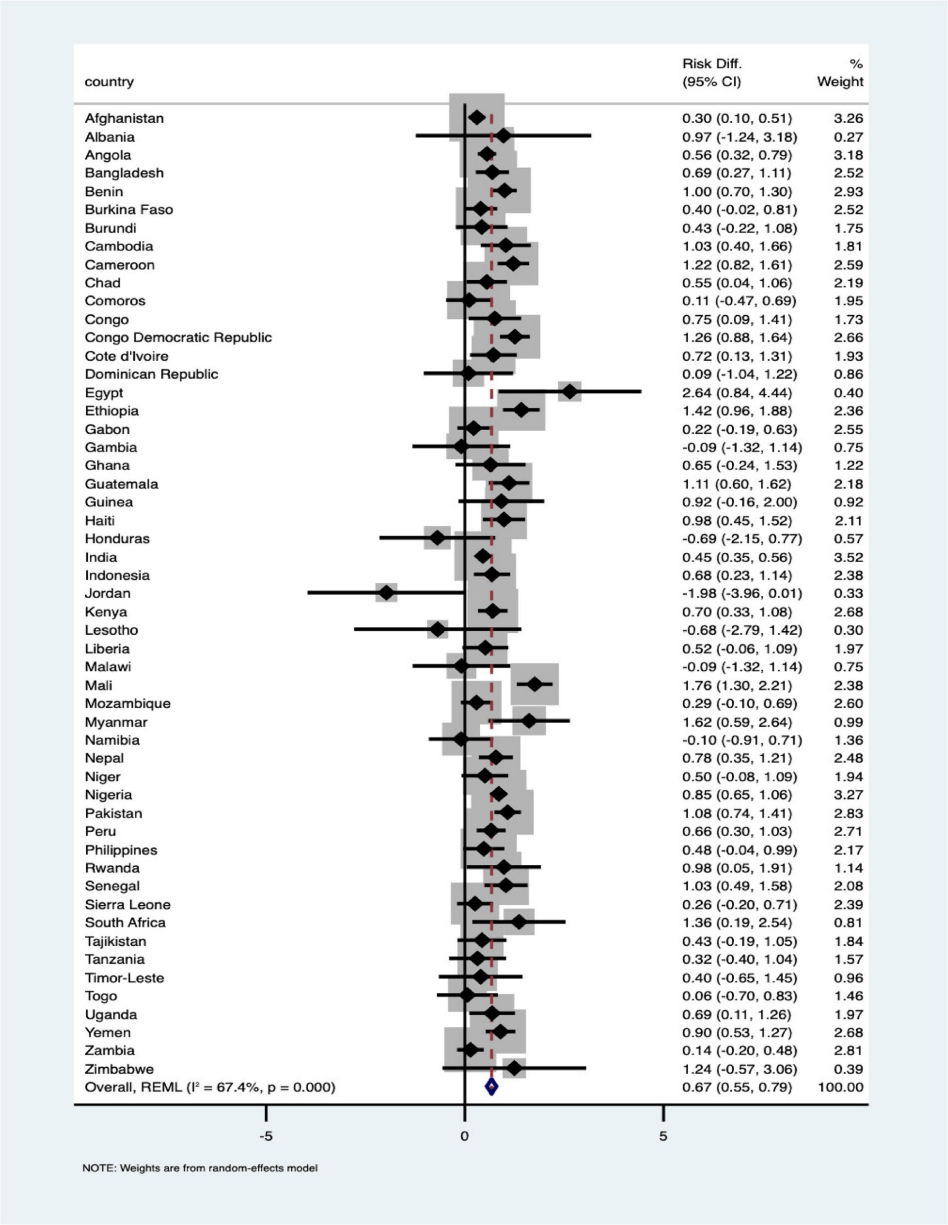
Unadjusted meta-analysis of suboptimal access to EHS among mothers-child pairs dwelling in slum and those in non-slum

As shown in Fig. 1, 52 of the 58 LMICs were included in the meta-analysis. Albania, Armenia, Colombia, Kyrgyz Republic, South Africa, and Yemen were excluded because of insufficient data to compute effect estimates. Significant pro-slum inequalities in suboptimal access to EHS were found in 28 of the 58 LMICs: Afghanistan, Angola, Bangladesh, Benin, Cambodia, Cameroon, Chad, Congo, Congo DR, Cote d’Ivoire, Egypt, Ethiopia, Guatemala, Haiti, India, Indonesia, Kenya, Mali, Myanmar, Nepal, Nigeria, Pakistan, Peru, Senegal, South Africa, Uganda, Yemen. In these countries, this indicates that suboptimal access to EHS was higher among mother–child pairs living in urban slums than those living in non-slums.

**Fig. 2:**
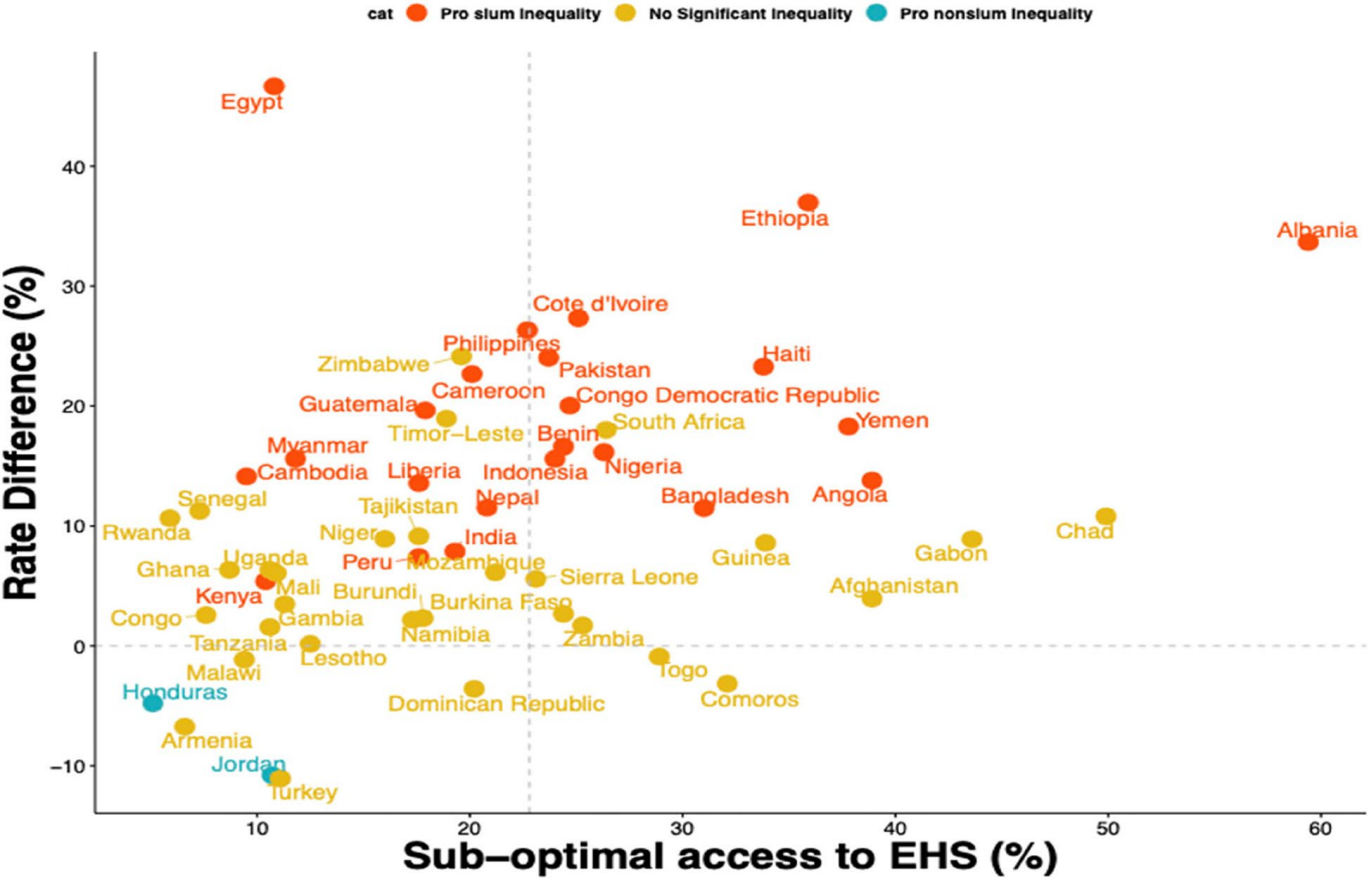
Scatterplot to map the distribution of urban pro-slum and non-slum inequalities in LMICs

In the sub-continent analysis (see Supplementary Fig. 1), of the 34 African countries included, 16 showed statistically significant pro-slum inequality in suboptimal access to EHS with the highest in Egypt and Mali (2.64[0.84–4.44] and 1.76 [1.65, 1.87] respectively). Three of the five LMICs included from the South American region showed significant pro-slum inequality with the highest and lowest risk in Guatemala (1.11[0.60, 1.62]) with urban women-child pairs in Honduras having insignificant non-slum inequalities (-0.69[2.55, 0.77]). Finally, pro-slum inequalities in suboptimal access to EHS was found in 9 of the 13 LMICs in Asia; the highest risk was found in Myanmar 1.62[0.59, 2.64] and insignificant non-slum inequalities in Jordan -1.98 [-3.96, 0.01]. Nevertheless, none of the LMICs showed a statistically significant non-slum inequality. Overall, mother–child pairs living in slums have an additional risk of 0.65(0.55–7.09). The chart in Fig. 2 was used to map the distribution of pro-slum and non-slum inequalities in LMICs with low and high suboptimal access to EHS.

**Fig. 3:**
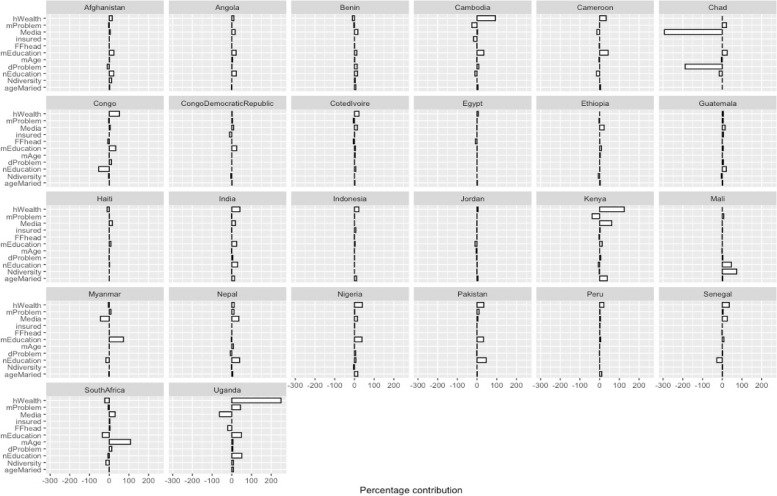
Plots from decomposition analysis showing the contribution of key variables to pro-slum inequality in access to EHS in LMICs

**Fig. 4:**
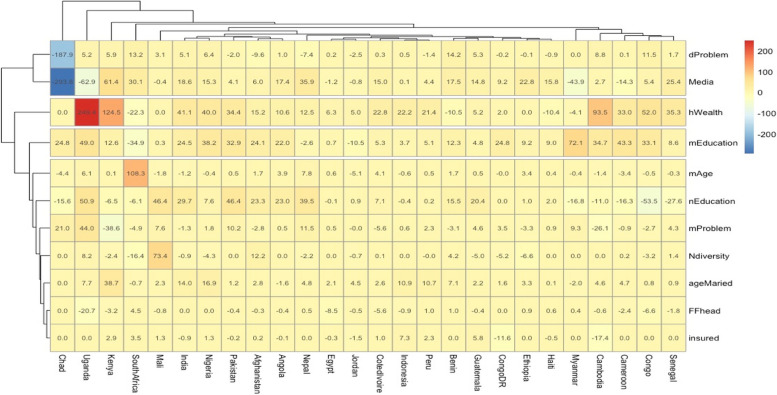
Heat map highlighting the contribution of key variables to pro-slum inequality in access to EHS

The decomposition analysis findings were presented in Figs. 3 and 4, showing how the covariates contributed to the inequality gaps in each LMIC. Of the 58 LMICs included in the study, only the 26 LMICs that showed pro-slum or non-slum inequalities were included in the decomposition analysis even though some were insignificant. There were apparent variations among the LMICs, but on average, household wealth, neighbourhood education level, and access to media and neighbourhood-level illiteracy contributed more to the inequality gaps in suboptimal access to EHS in LMIC’s slums. Household wealth contributed most to the inequality gap in Uganda and substantially in Cambodia, Cameroon, Congo, India, Indonesia, Kenya, Nigeria, Nigeria, and Pakistan. Meanwhile, money problems to access health were insignificant in some countries such as India, Indonesia, and Congo. Access to media reduced the inequality gaps in Cambodia, Chad, Kenya, Myanmar, Uganda, and mother’s age at birth was the most important contributory covariate in South Africa. Neighbourhood diversity was significant only in Mali.

## Discussion

This study set out to examine the inequality in access to EHS associated with urban slum and non-slum residences using individual-level meta-analysis and decomposition analysis. The former was done to identify countries with significant inequalities, while the latter was performed to highlight the explained and unexplained factors associated with suboptimal access to EHS. Findings showed a wide variation in suboptimal access to EHS between mother–child pairs residing in urban slums and non-slums. These findings might reflect the challenges in expanding EHS in LMICs with a high population living in slums.

Table 3 showed that the pooled average rate of suboptimal access EHS among mother–child pairs dwelling in urban slums was 32.7% against 20% for pairs residing in non-slums. However, the differences in the inter-country percentage of mother–child pairs dwelling in slums were substantial. Going by the definition in this study, Liberia and Congo DR have the highest, 73.6% and 63.0%, respectively while Egypt had the lowest. The 2018 World Bank estimates corroborated the findings from this study as they reported that 70% of urban dwellers in Liberia live in slums [19]. This has been linked to the widespread of the Ebola virus in the country between 2013–2016; a similar high prevalence of urban slum residence in the Congo Republic (about 50%) has been reported [11, 20]. Differences between our numbers and the World Bank are likely due to the differing and stricter definitions of slum residence applied in this study.

Findings in Table 1 and Fig. 1 highlight countries where mother–child slum residence are strongly associated with access to EHS. LMICs such as Afghanistan, Nigeria, Bangladesh, Comoros, Ethiopia, India, and Mozambique had a high prevalence of urban slum residences with a huge impact on access to EHS; the impacts in some other countries were even larger. Egypt has the lowest prevalence (less than 1%) of mother–child pairs dwelling in urban slums; however, the highest impact of slum residence on access to EHS was observed in Egypt. Countries such as Haiti, Indonesia, Pakistan, Zimbabwe, and South Africa showed a similar pattern to Egypt, having a low prevalence of slum residences but with a huge impact on access to EHS. Using slum residences as a surrogate indicator for the neighbourhood effect, these findings support the hypothesis that neighbourhood or contextual factors have a significant association with people’s access to EHS. It is intuitive to deduce that this association is linked to the established facts that slum dwellers have limited access to facilities, resources, and health services [21, 22]. Slums across several LMICs are characterised and faced by similar challenges such as overcrowding and poor sanitation even where the facilities and services are in existence [21, 23]. These findings support the current call at the global level for increased attention to tackling informal settlements and rural–urban migrations as strategies to improve urban health.

From the decomposition analysis, household wealth, maternal education level, and access to media were the major contributors to the inequality gaps in access to EHS across all the included countries. These determinants are interconnected as previous research studies have shown that household wealth status has a positive relationship with educational attainment and access to media [24–26]. More importantly, these findings further suggest that drivers of poor access to EHS are similar across different settings in most LMICs. This has the potential to help policymakers and stakeholders focus their programming efforts aimed at driving UHC goals in LMICs.

A few countries showed some variations that are noteworthy to mention. For example, in South Africa, the mother’s age is the most significant contributor to the urban slum non-slum inequality gap in access to EHS. The rate of maternal and under-five mortality has been declining in the past few years but has plateaued recently [27]. The findings from this study might provide some insight into how to combat these issues for further decline. In Mali, the neighbourhood level of diversity is the most substantial contributor to the inequality gap; this finding deviates from previous literature as diversity is believed to be a driving factor to advance UHC goals, including coverage of EHS [28].

Beyond the known pathway of association between slum residence, access to health and health outcomes, some studies have explored the role of social networks in slum residence and its impact on health-seeking behaviour and health outcomes. A recent systematic review by Onegbu and colleagues [29] reported the frequent use of lay consultation (health advice seeking amongst personal networks) among slum dwellers and how it positively and negatively affects health-seeking behaviour and adherence to medical expert advice. At the peak of Ebola virus infection in countries like Liberia, social networks in slums were identified as one of the strongest means of receiving relevant information – both misinformation and those based on evidence [20, 30]. While countries continue to tackle issues related to informal settlements to reduce slum residences, health programmers and policymakers can leverage this potential of social networks to spread suitable and evidence-based health information.

## Conclusion

Understanding the impact of neighbourhood effect on access to EHS and overall health outcomes for the population in specific neighbourhood settings is critical for designing evidence-based and contextually relevant interventions. This study revealed a wide variation in suboptimal access to EHS between mother–child pairs residing in urban slums and non-slums. Specifically, factors such as household wealth, maternal and neighbourhood education levels, and access to media were identified as the major contributors to the inequality gaps in EHS access across all the included countries. To sustainably achieve UHC goals and improve EHS access, key public health policy and programme stakeholders should consider these factors that facilitate neighbourhood-related inequality gaps in access to EHS and drive needed transformative interventions, including targeting the urban poor. Our study is not without limitations, due to the study design being cross-sectional, it’s important to note that the significant association observed in our study does not equate to causality.

### Supplementary Information

Supplementary Material 1.

## Data Availability

Data for this study were from the Demographic and Health surveys (DHS) and available here: http://dhsprogram.com/data/available-datasets.cfm.
