# Supplementary material for: Neighbourhood effect and inequality in access to essential health services among mother–child paired samples: a decomposition analysis of data from 58 low- and middle-income countries
Source: Int J Equity Health. 2024 Jun 28;23:130. doi: 10.1186/s12939-024-02194-4 (PMC11212233; doi:10.1186/s12939-024-02194-4)
Supplement: Supplementary file 1 — Supplementary Material 1. [file 12939_2024_2194_MOESM1_ESM.docx]

S Fig 1: Unadjusted sub-country meta-analysis of suboptimal access to EHS among mothers-child pairs dwelling in slum and those in non-slum.
